# Berberine Relieves Metabolic Syndrome in Mice by Inhibiting Liver Inflammation Caused by a High-Fat Diet and Potential Association With Gut Microbiota

**DOI:** 10.3389/fmicb.2021.752512

**Published:** 2022-01-12

**Authors:** Jinjin Li, Jialin Li, Jiajia Ni, Caibo Zhang, Jianlei Jia, Guoying Wu, Hongzhao Sun, Shuzhen Wang

**Affiliations:** ^1^School of Life Sciences, Qilu Normal University, Jinan, China; ^2^Jinan Zhangqiu District Hospital of Traditional Chinese Medicine, Jinan, China; ^3^Research and Development Center, Guangdong Meilikang Bio-Science Ltd., Dongguan, China; ^4^Dongguan Key Laboratory of Medical Bioactive Molecular Developmental and Translational Research, Guangdong Medical University, Dongguan, China

**Keywords:** berberine, metabolic syndrome, gut microbiota, high-fat diet, obesity

## Abstract

Whether berberine mediates its anti-inflammatory and blood sugar and lipid-lowering effects solely by adjusting the structure of the gut microbiota or by first directly regulating the expression of host pro-inflammatory proteins and activation of macrophages and subsequently acting on gut microbiota, is currently unclear. To clarify the mechanism of berberine-mediated regulation of metabolism, we constructed an obese mouse model using SPF-grade C57BL/6J male mice and conducted a systematic study of liver tissue pathology, inflammatory factor expression, and gut microbiota structure. We screened the gut microbiota targets of berberine and showed that the molecular mechanism of berberine-mediated treatment of metabolic syndrome involves the regulation of gut microbiota structure and the expression of inflammatory factors. Our results revealed that a high-fat diet (HFD) significantly changed mice gut microbiota, thereby probably increasing the level of toxins in the intestine, and triggered the host inflammatory response. The HFD also reduced the proportion of short-chain fatty acid (SCFA)-producing genes, thereby hindering mucosal immunity and cell nutrition, and increased the host inflammatory response and liver fat metabolism disorders. Further, berberine could improve the chronic HFD-induced inflammatory metabolic syndrome to some extent and effectively improved the metabolism of high-fat foods in mice, which correlated with the gut microbiota composition. Taken together, our study may improve our understanding of host-microbe interactions during the treatment of metabolic diseases and provide useful insights into the action mechanism of berberine.

## Introduction

With a growing increase in changes in diet, lifestyle and other factors, there has been an explosive epidemic trend of metabolic syndrome characterized by obesity, type II diabetes, non-alcoholic fatty liver disease (NAFLD), and atherosclerosis (Ley et al., 2006; Turnbaugh et al., 2006; Zhang H. et al., 2010). As one of the most serious diseases threatening public health (Haslam and James, 2005; Moustafa and Froguel, 2013; Hall et al., 2015), metabolic syndrome can also cause other diseases, such as NAFLD, which can further develop into steatohepatitis, liver fibrosis, cirrhosis, liver cancer (Osborn and Olefsky, 2012; Yoshimoto et al., 2013; Ni et al., 2021), diabetes, or hypertension and cause multiple organ damage (Thandavarayan et al., 2011; Mozaffari-Khosravi et al., 2012; Wada et al., 2012). Although various drugs are available to treat metabolic syndrome and its complications in the clinical setting, there is presently no specific treatment for this condition (Zhang et al., 2012, 2015; Chang et al., 2015). For example, although metformin and acarbose can reduce blood sugar, its gastrointestinal side effects make it impossible for many patients to tolerate the maximum dose. While thiazolidinediones (TZDs) can reduce insulin resistance, it also causes weight gain and hypoglycaemia (Srinivasan et al., 2015). Therefore, exploring the methods and mechanisms for prevention and treatment of metabolic syndrome has important theoretical and clinical application value.

Metabolic syndrome is typically accompanied by low-level, systemic chronic inflammation, leading to impaired insulin activity and the development of metabolic abnormalities (Boulangé et al., 2016; Reilly and Saltiel, 2017). It has been reported that the activation of inflammation effector molecules contributes to the desensitization of insulin signaling pathways (Ribes-Navarro et al., 2019). At the molecular level, the activation of IκB kinase complex, extracellular signal-regulated protein kinases 1 and 2 (ERK1/2), and c-Jun N-terminal kinases (JNKs) in the inflammatory tissues of obese individuals can reduce insulin receptor substrate (IRS) protein tyrosine phosphorylation levels, leading to the attenuation of the insulin signal (Tanti et al., 2012). Furthermore, the production of cytokines, such as tumor necrosis factor alpha (TNF-α), in rodent and human visceral adipose tissues regulates insulin sensitivity by altering the expression levels of genes encoding insulin receptor 1 (IRS-1), glucose transporter GLUT4, and PPAR-α (Larsen et al., 2007; Tack et al., 2012). In turn, the loss of insulin sensitivity can trigger fasting hyperglycemia and increase liver lipid synthesis and fat accumulation (Saltiel and Kahn, 2001; Delarue and Magnan, 2007). These responses enhance chronic low-grade inflammation, induce the recruitment and activation of various mature immune cells (including mast cells, macrophages, and dendritic cells) and adipocytes in metabolic tissues, and ultimately, further augment the inflammatory response (Lumeng and Saltiel, 2011; Sell et al., 2012).

Various microorganisms inhabit the intestines of vertebrates. Cooperatively, the gut microbiota play an important role in the growth, development, metabolism, and immunity of the host (Turner, 2009; Levy et al., 2017; Cani et al., 2019; Seth et al., 2019; Ruff et al., 2020). Additionally, recent studies have demonstrated that gut microbes play an important role in the occurrence and development of metabolic diseases such as obesity, insulin resistance, atherosclerosis, and NAFLD (Cani et al., 2007). Gut microbiota help break down hard-to-digest food components, such as dietary fiber (Qin et al., 2010), and can also regulate the metabolism of bile acids, lipids, and amino acids in the host by exchanging metabolites with the host and participating in signal pathways and regulating the host gene expression and energy homeostasis (Bäckhed et al., 2004; Turnbaugh et al., 2006; Velagapudi et al., 2010). However, structural imbalance of the gut microbiota due to high-fat diet (HFD) intake may damage the intestinal barrier, thereby leading to increased levels of inflammatory factors and lipopolysaccharides (LPS) in the circulatory system, which can trigger metabolic inflammation, and subsequently, induce insulin resistance, obesity, and diabetes (Cani et al., 2008). Moreover, gut microbiota imbalance, particularly a reduction in the abundance of *Bifidobacterium spp*. that protect the intestinal barrier function and an increase in the abundance of pathogenic bacteria such as *Desulfovibrio spp*. that produce endotoxin and H_2_S, can compromise the barrier function of the intestine (Zhang et al., 2008). This causes high levels of intestinal toxins such as LPS to enter the circulatory system of the host and induce chronic systemic low-grade inflammation, a condition known as “metabolic endotoxemia.” Eventually, long-term chronic inflammation can lead to a series of metabolic diseases such as decreased insulin sensitivity and NAFLD (Yin et al., 2008a).

Berberine hydrochloride exhibits a modest inhibitory effect on bacteria, fungi, and viruses. Berberine is mainly used clinically to treat intestinal infections and bacterial diarrhea (Hwang et al., 2003; Villinski et al., 2003). In addition, berberine can also be used to treat various metabolic diseases such as diabetes, hyperlipidemia, inflammation, and cardiovascular disease (Li et al., 2008; Yin et al., 2008a,b; Zhang et al., 2008; Zhang C. H. et al., 2010). The anti-inflammatory effects of berberine are primarily linked to the regulation of blood lipids, inhibition of oxidative stress, reduction of cell apoptosis, regulation of cell energy metabolism and inflammatory factor expression, and increased adiponectin levels (Wu and Wang, 2008; Wu et al., 2010). Furthermore, berberine can inhibit the activation and transcription of inflammatory cytokines by suppressing the activation of nuclear factor-kappa B (NF-κB), thus displaying activity against metabolic syndrome (Zhu et al., 2018). For example, berberine can reduce the expression of TNF-α, IL-6, and leptin in 3T3-L1 adipocytes (Choi et al., 2006). In addition, berberine plays a role in reducing insulin resistance by inhibiting the phosphorylation of IKKβ (Ser181) and the translocation of NF-κB (P65) into the nucleus (Yi et al., 2008). Since the oral bioavailability of berberine is very low and most of the drug is not absorbed and excreted intact in feces, a major part of the drug can directly interact with the gut microbiota (He et al., 2009). Studies have shown that the gut microbiota-derived metabolites of berberine can inhibit the entry of NF-κB (P65) into the nucleus by downregulating the expression of TLR4 and MyD88, which in turn inhibits the TLR4-MyD88-NF-κB signaling pathway. This indicates that colitis can be alleviated by reducing the expression of related inflammatory factors such as TNF-α, NF-κB, IL-6, and inducible nitric oxide synthase (iNOS; Li et al., 2020). It has been reported that berberine intervention in hamsters with HFD-induced obesity inhibits weight gain and fat deposition; reduces blood sugar, blood lipid, and cellular lipopolysaccharide levels; improves inflammation and insulin resistance; and relieves fatty liver disease and leads to increased production of short chain fatty acids (SCFA; Guo et al., 2019). This indicates that the metabolic regulation mechanism of berberine is likely closely related to the gut microbiota status, which represents a potential target for the development of therapeutic drugs. However, whether berberine mediates its anti-inflammatory and blood sugar and lipid lowering effects solely by adjusting the structure of the gut microbiota or by first directly regulating the expression of host pro-inflammatory proteins and activating macrophages and then acting on gut microbiota, is currently unclear. The answer to this question will help clarify the anti-inflammatory mechanism of berberine and provide theoretical guidance for the subsequent development of berberine and antibiotics, or berberine and probiotics for the treatment of metabolic syndrome.

Accordingly, this study used SPF-grade C57BL/6J male mice to construct an obese mouse model and conduct a systematic study on liver tissue pathology, inflammatory factor expression, and gut microbiota structure. We screened the targets of berberine in gut microbiota and showed that the molecular mechanism of berberine activity against metabolic syndrome involves the regulation of gut microbiota structure and expression of host inflammatory factors.

## Materials and Methods

### Animal Models and Experimental Design

**FIGURE 1:**
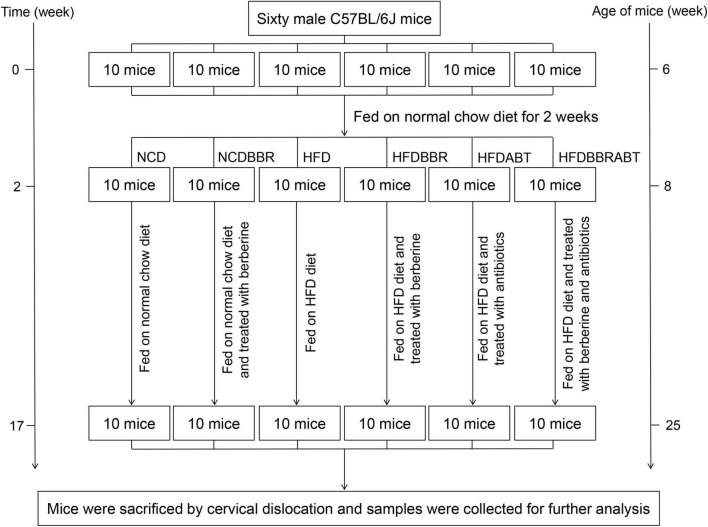
Framework shows the main process of the experiment. NCD, normal chow diet; NCDBBR, NCD supplemented with berberine; HFD, high fat diet; HFDBBR, HFD supplemented with berberine; HFDABT, HFD supplemented with antibiotics (by drinking freely); HFDBBRABT, HFD supplemented with berberine and antibiotics. Antibiotic-treated mice were fed water containing 1 g/L ampicillin, 0.5 g/L vancomycin, 1 g/L neomycin, and 1 g/L metronidazole.

Animal experiments were approved by and performed in accordance with the guidelines of the Animal Ethics Committee of the Qilu Normal University. Sixty male C57BL/6J mice (6 weeks old) were obtained from the Laboratory Animal Center of Shandong Academy of Medical Sciences (Jinan, China) and fed on normal chow diet for 2 weeks to adapt to the environment. Thereafter, animals were divided into six groups (*n* = 10 per group) as follows: Normal chow diet (NCD group; 10% kcal from fat, Open Source Diets, D12450B), NCD supplemented with berberine [100 μg/kg/d body weight was suspended in 0.5% sodium carboxymethyl cellulose (CMC-Na) solution and fed through gavage; NCDBBR group], high-fat diet (HFD group; 60% kcal from fat, Open Source Diets, D12492), HFD supplemented with berberine (100 μg/kg/d body weight was suspended in 0.5% CMC-Na solution and fed through gavage; HFDBBR group), HFD supplemented with antibiotics (HFDABT group), and HFD supplemented with berberine and antibiotics (HFDBBRABT group). The mice were treated for 15 weeks, and drank freely during the experiment (Figure 1). To control the influence of the solvent, the same volume of 0.5% CMC-Na used to dissolve berberine was also administered to the NCD and HFD groups. To analyze whether berberine could still reduce fat accumulation and inhibited liver inflammation in mice fed with high-fat diet when the gut microbiota was inhibited by antibiotics, we set two antibiotic-treated groups (i.e., HFDABT and HFDBBRABT). Antibiotic-treated mice were fed water containing 1 g/L ampicillin, 0.5 g/L vancomycin, 1 g/L neomycin, and 1 g/L metronidazole, all of which were obtained from Solarbio (Beijing, China) (Kamilla et al., 2017). Mice were housed in standard plastic cages (five mice per cage) and maintained under a 12 h light-dark cycle at constant temperature and humidity [(23 ± 1°C) and (55 ± 5%), respectively]. To record mice body weight, six mice in each group (five mice in the HFDABTBBR group) were randomly weighed weekly at the end of each week.

### Sample Collection

Mice were sacrificed by cervical dislocation and fixed on a dissecting plate. The abdominal cavity was quickly opened and the liver gently removed. After rinsing with phosphate-buffered saline (PBS), some liver tissues were fixed with 10% neutral buffered formalin for tissue section analysis and the rest were quickly frozen in liquid nitrogen and stored at −80°C until used for RT-PCR analysis. Five replicates of fecal samples were randomly collected from each group of mice and stored at −80°C for subsequent sequence analysis.

### Insulin Sensitivity

Oral glucose tolerance test (OGTT) and insulin tolerance test (ITT) were performed at the 16th week. Three mice in each group were randomly collected to OGTT and another three mice were used to ITT. Before the OGTT, mice were fasted for 8 h and then administered 2 g/kg body weight glucose orally. Blood glucose levels (BGL) were determined with a glucose meter (Yuwell Medical Equipment Co., Ltd., Jiangsu, China) after 0, 15, 30, 60, 90, 120, and 150 min. Before the ITT, mice were fasted for 4 h and insulin (0.75 U/kg) was injected intraperitoneally. Blood glucose levels were determined, as described above, after 0, 15, 30, 60, 90, 120, and 150 min.

### Histopathological Analysis

Liver tissue was fixed in 10% neutral buffered formalin; then, it was processed routinely, embedded in paraffin wax, sectioned (4 μm thickness), and stained with hematoxylin and eosin (H&E). Histopathological assessment was conducted using a BX53 + DP26 light microscope (Olympus, Japan) (Lin et al., 2019). Oil Red staining was used to visualize the fat in the tissue, and hematoxylin was used to stain the nucleus. F4/80 immunohistochemical detection was performed to determine the distribution of macrophages in mouse liver.

### Quantitative Reverse Transcription-Polymerase Chain Reaction

Total RNA was isolated from liver samples using the One-step RT-PCR Kit (TOYOBO, China) according to the manufacturer’s protocol. The concentration of total RNA was determined using a NanoDrop One spectrophotometer (Thermo Fisher Scientific, United States). Then, 1 μg of total RNA was reverse transcribed into cDNA using oligo(dT) primers. Next, qRT-PCR was performed to analyze gene expression using the SYBR Green PCR Master Mix (Tiangen, Beijing, China). The transcriptional levels of target genes were normalized against the mRNA levels of glyceraldehyde-3-phosphate dehydrogenase (*GAPDH*). Primers for target genes, listed in Table 1, were designed using the Oligo 7.0 Program. The primers were synthesized by the Shanghai Shenggong Biotechnology Co., Ltd. The SYBR^®^ Premix Ex Taq™ was purchased from Takara Biomedical Technology (Beijing) Co., Ltd. The amplification reactions were carried out on a LightCycler 480 Instrument (Roche Diagnostics GmbH) with an initial hold step (95°C for 10 min) and 45 cycles of a three-step PCR (95°C for 10 s, 60°C for 20 s, and 72°C for 30 s). After verifying that the amplification efficiencies of the selected genes and GAPDH were approximately equal, differences in expression levels were calculated using the 2^–ΔΔCt^ method (Livak and Schmittgen, 2001).

**TABLE 1:** Sequences of primers used for qRT-PCR.

Name	Primer sequence (5′→3′)
*mGAPDH-F*	*AGG TCG GTG TGA ACG GAT TTG*
*mGAPDH-R*	*TGT AGA CCA TGT AGT TGA GGT CA*
*mTnfα-F*	CAGGCGGTGCCTATGTCTC
*mTnfα-R*	CGATCACCCCGAAGTTCAGTAG
*mCox-2-F*	ACGGTCCTGAACGCATTTATG
*mCox-2-R*	TTGGCCCCATTTAGCAATCTG
*mReg3g-F*	ATGCTTCCCCGTATAACCATCA
*mReg3g-R*	ACTTCACCTTGCACCTGAGAA
*miNOS-F*	GTTCTCAGCCCAACAATACAAGA
*miNOS-R*	GTGGACGGGTCGATGTCAC
*mArg1-F*	TGGCTTGCGAGACGTAGAC
*mArg1-R*	GCTCAGGTGAATCGGCCTTTT

### Gut Microbiota Composition Analysis

Genomic DNA was extracted using the PowerFecal DNA Kit according to manufacturer’s protocols (QIAGEN, Germany). The V4–V5 hypervariable region of prokaryotic 16S rRNA gene was amplified using the universal primers 515F and 909R as previously described (Xiang et al., 2018; Ni et al., 2019). The amplicons were quantified with a NanoDrop 2000 spectrophotometer, and equimolar amounts of each sample were pooled and purified using the AxyPrep DNA gel extraction kit (Axygen, China). The purified DNA fragments were sequenced using an Illumina MiSeq system at Guangdong Meilikang Bio-Science Ltd., China.

Raw reads were merged using the FLASH 1.2.8 software (Magoc and Salzberg, 2011) and processed using the QIIME 1.9.0 pipeline (Caporaso et al., 2010), as previously described (Ni et al., 2017; Huang et al., 2018). Briefly, all merged sequences were trimmed and assigned to each sample based on their barcode sequences with no mismatch. Low-quality and chimera sequences were removed. Subsequently, the sequences were randomly resampled to obtain the same number of sequences in each sample. Then, high-quality sequences were clustered into operational taxonomic units (OTUs) at 97% identity using the UPARSE software (Edgar, 2013). Each OTU was assigned to taxonomic groups using the RDP classifier (Wang et al., 2007) with gg_13_8 dataset. Functional profiles of gut microbiota were predicted using the phylogenetic investigation of communities by reconstruction of unobserved states (PICRUSt; Langille et al., 2013).

### Data Analysis

One-way analysis of variance (ANOVA) with Tukey–Kramer *post hoc* test was conducted using R 2.5.1 (R Core Team, 2013). The Kruskal–Wallis *H*-test with Welch’s *post hoc* test was conducted using the Statistical Analysis of Metagenomic Profiles (STAMP) software (Parks et al., 2014). Non-parametric multivariate analysis of variance (PERMANOVA) (Anderson, 2001) was conducted using the vegan package (Dixon, 2003) in R 3.5.1 (R Core Team, 2013). Kruskal–Wallis *H*-tests to screen significantly different taxa were conducted using the STAMP software (Parks et al., 2014). Principal co-ordinates analysis (PCoA) was conducted using the QIIME 1.9.0 pipeline (Caporaso et al., 2010), and a heatmap profile was drawn using the pheatmap package in R. Differences with *p* < 0.05 were considered significant.

## Results

### Berberine Significantly Alleviates the Weight Gain and Insulin Resistance Induced by a High-Fat Diet

**FIGURE 2:**
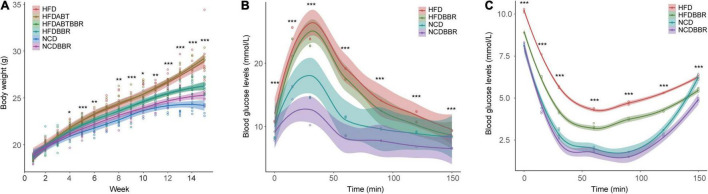
Changes in body weight **(A)** and blood glucose levels of mice detected using oral glucose tolerance test (OGTT) **(B)**, and insulin tolerance test (ITT) **(C)**. HFD, high fat diet group; HFDBBR, HFD supplemented with berberine; HFDABT, HFD supplemented with antibiotics (by drinking freely); HFDABTBBR, HFD supplemented with berberine and antibiotics; NCD, normal chow diet; and NCDBBR, NCD supplemented with berberine. *p < 0.05; **p < 0.01; ***p < 0.001. Five replicates are represented by the data.

The body weight of HFD mice was significantly higher than that of NCD mice (one-way ANOVA, *p* < 0.05), whereas the body weight of HFDBBR mice was significantly lower than that of HFD mice in the end of the experiment (Figure 2A and Supplementary Figure 1A). These results indicated that berberine could effectively reduce the weight gain caused by high-fat food. Antibiotic treatment of the mice fed with high-fat diet (HFDABT group) did not significantly decrease the weight gain in mice relative to that in the HFD group (Figure 2A and Supplementary Figure 1A). The combination of antibiotics and berberine (HFDABTBBR) had the same effect as berberine alone in inhibiting weight gain caused by high-fat diet (Figure 2A and Supplementary Figure 1A).

Oral glucose tolerance test and ITT results showed that at the end of the experiment, the blood glucose levels of HFD mice were significantly (*P* < 0.05) higher than those of the NCD mice, whereas the blood glucose levels of HFDBBR mice were lower than those of HFD mice (Figures 2B,C and Supplementary Figures 1B,C). Moreover, the blood glucose levels of NCD mice treated with berberine were significantly lower than those of NCD mice without berberine treatment (Figures 2B,C and Supplementary Figures 1B,C). These results indicated that berberine could significantly reduce the blood glucose levels and alleviate the insulin resistance in mice.

### Berberine Reduces Fat Accumulation by Inhibiting Liver Inflammation in Mice

**FIGURE 3:**
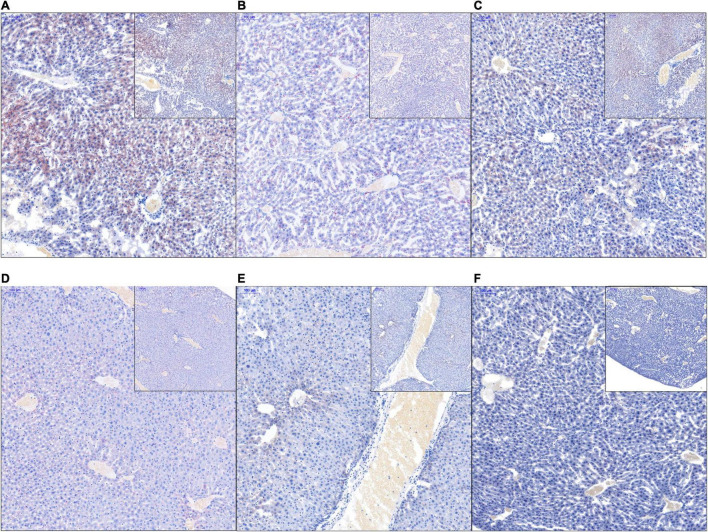
Liver pathological tissue section stained with hematoxylin and eosin and oil red. Red stain indicates adipocytes. **(A)** HFD; **(B)** HFDABT; **(C)** HFDBBR; **(D)** HFDABTBBR; **(E)** NCD; **(F)** NCDBBR. HFD, high fat diet group; HFDBBR, HFD supplemented with berberine; HFDABT, HFD supplemented with antibiotics (by drinking freely); HFDABTBBR, HFD supplemented with berberine and antibiotics; NCD, normal chow diet; and NCDBBR, NCD supplemented with berberine. The upper-right of each histologic photo is a histologic photo with a larger scale.

Histochemical analysis of liver tissue sections using Oil Red staining revealed the presence of a large number of red-stained adipocytes in the livers of mice in the HFD group, but not in those of the HFDBBR group (Figure 3). Treatment with compound antibiotics (HFDABT and HFDABTBBR groups) evidently decreased accumulation of red-stained adipocytes in the livers of mice in the HFD group (Figures 3B,D). These results implied that gut microbiota derived by high-fat diet probably promoted the accumulation of fat in the liver, both berberine and compound antibiotics could relieve the accumulation.

**FIGURE 4:**
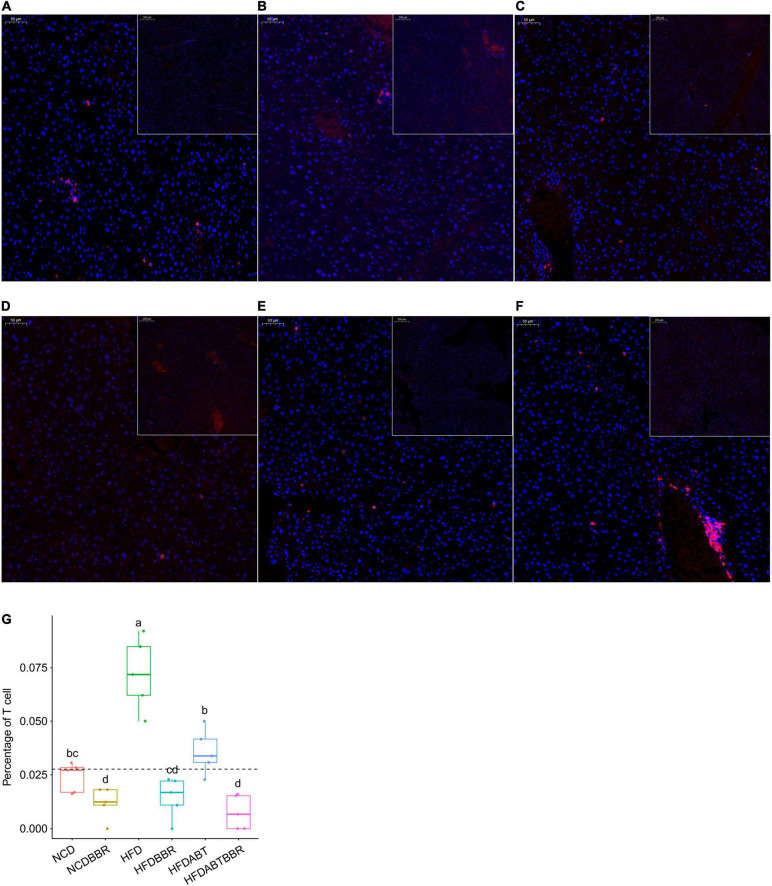
F4/80 immunohistochemical analysis of the effect of berberine on the number of liver macrophages in mice fed an HFD. Blue stain indicates the nucleus, whereas brown stain marks macrophages. **(A)** HFD; **(B)** HFDABT; **(C)** HFDBBR; **(D)** HFDABTBBR; **(E)** NCD; **(F)** NCDBBR. **(G)** Boxplots showing significant differences in the percentage of macrophages in the livers of mice in various diet groups. HFD, high fat diet group; HFDBBR, HFD supplemented with berberine; HFDABT, HFD supplemented with antibiotics (by drinking freely); HFDABTBBR, HFD supplemented with berberine and antibiotics; NCD, normal chow diet; and NCDBBR, NCD supplemented with berberine. Different letters above the boxplots indicate significant differences. The upper-right of each histologic photo is a histologic photo with a larger scale.

**FIGURE 5:**
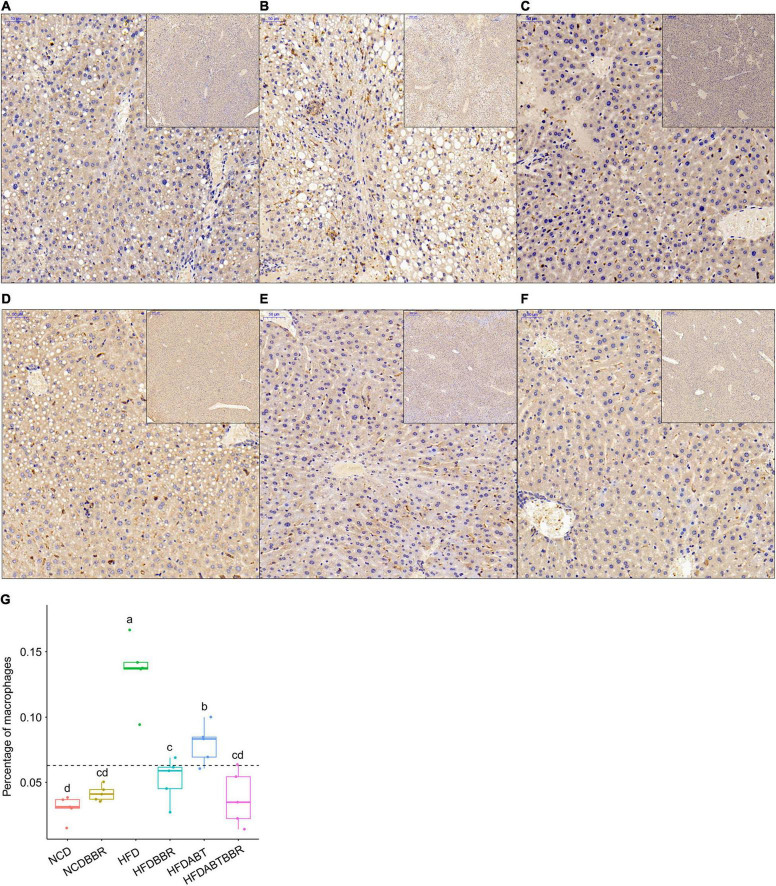
CD4 immunofluorescence detection showing the effect of berberine on the number of T cells in the livers of HFD mice. Blue stain indicates the nucleus, whereas red stain labels T cells. **(A)** HFD; **(B)** HFDABT; **(C)** HFDBBR; **(D)** HFDABTBBR; **(E)** NCD; **(F)** NCDBBR. **(G)** Boxplots showing significant differences in the percentage of T cells. HFD, high fat diet group; HFDBBR, HFD supplemented with berberine; HFDABT, HFD supplemented with antibiotics (by drinking freely); HFDABTBBR, HFD supplemented with berberine and antibiotics; NCD, normal chow diet; and NCDBBR, NCD supplemented with berberine. Different letters above the boxplots indicate significant differences. The upper-right of each histologic photo is a histologic photo with a larger scale.

Macrophages and T cells are important immune cells to study phagocytosis, cellular immunity, and molecular immunology (Gordon, 2016; Peterson et al., 2018). Moreover, macrophage recruitment is closely related to the occurrence of fatty liver (Hill et al., 2014; Gordon and Pluddemann, 2017). Our results showed that the number of macrophages, which stained brown, was significantly higher in the liver of the HFD group than that in the liver of the NCD group. However, the numbers of macrophages in the HFDBBR and HFDABT groups were significantly reduced (one-way ANOVA, *p* < 0.05; Figure 4). This result indicated that both berberine and compound antibiotics reduced the number of macrophages in the livers of mice fed with an HFD. Analysis of the distribution of T cells in the liver via CD4 immunofluorescence detection revealed that T-cell infiltration in the HFD group was significantly higher than that in the NCD group (one-way ANOVA, *p* < 0.05; Figure 5), indicating increased inflammation. In contrast, T-cell infiltrations in the HFDBBR and HFDABT groups were significantly lower than that in the HFD group (one-way ANOVA, *p* < 0.05; Figure 5), indicating that both berberine and compound antibiotics could significantly reduce the liver inflammation caused by HFD.

**FIGURE 6:**
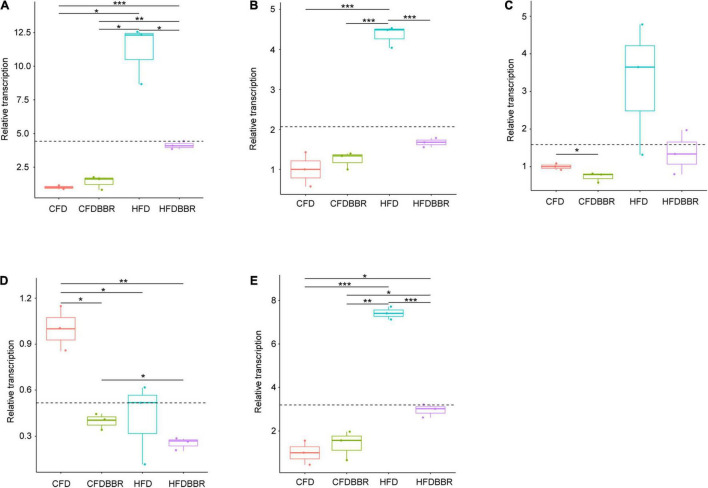
Expression levels of inflammatory factors in liver tissues of mice **(A)** TNF-α; **(B)** cox-2; **(C)** Reg3g; **(D)** iNOS; and **(E)** Arg1. The expression levels were determined by qRT-PCR and normalized to mRNA levels of GAPDH. HFD, high fat diet group; HFDBBR, HFD supplemented with berberine; NCD, normal chow diet; and NCDBBR, NCD supplemented with berberine. *p < 0.05; **p < 0.01; ***p < 0.001.

Gene expression analysis via qRT-PCR showed that the transcription levels of *TNF-α* and *cox-2* in the HFD group were significantly higher than those in the NCD group (one-way ANOVA, *p* < 0.05; Figures 6A,B). Although the transcription levels of *Reg3γ* in the HFD group were not significantly different from those in the NCD group (one-way ANOVA, *p* > 0.05), it still showed an increasing trend (Figure 6C). The transcription levels of *TNF-α* and *cox-2* in the livers of the HFDBBR group of mice treated with berberine were significantly lower than those in the HFD group (one-way ANOVA, *p* < 0.05). However, the transcription levels of *TNF-α* in HFD mice were higher than those in NCD mice (Figures 6A,B). Although the transcription levels of *iNOS* are usually significantly upregulated during liver inflammation (Menon and Sudheer, 2007), our results showed that HFD and berberine intervention significantly reduced *iNOS* expression levels (one-way ANOVA, *p* < 0.05; Figure 6D).

To detect the polarization direction of macrophages, we tested the expression of related inflammatory factors following macrophage polarization. The results showed that the *Arg1* factor, which represents M2 polarization, was significantly upregulated in the HFD group. However, its expression was significantly reduced in the HFDBBR group (one-way ANOVA, *p* < 0.05), indicating that berberine treatment biased macrophages toward M2 polarization (Figure 6E). Therefore, we speculated that berberine might improve hepatic fatty inflammation by influencing the polarization direction of macrophages.

### Berberine Relieves the Disruption in Gut Microbiota Structure Caused by an High-Fat Diet

**FIGURE 7:**
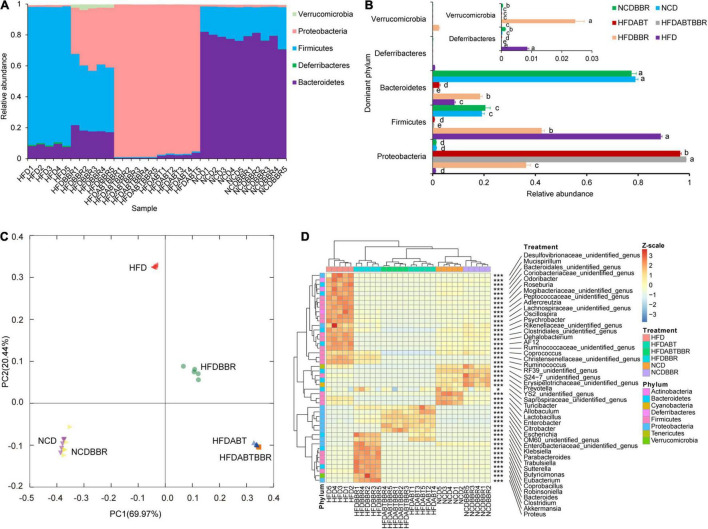
Effects of diet type, berberine, and antibiotic treatment on the composition of gut microbiota in mice. **(A)** Proportion of dominant phyla; **(B)** Influence of different diets and berberine and antibiotic treatments on the relative abundance of the dominant phyla; **(C)** PCoA ranking results based on OTU composition; **(D)** Changes in the relative abundance of dominant genera of gut microbiota in mice under different diet and berberine and antibiotic treatments. HFD, high fat diet group; HFDBBR, HFD supplemented with berberine; HFDABT, HFD supplemented with antibiotics (by drinking freely); HFDABTBBR, HFD supplemented with berberine and antibiotics; NCD, normal chow diet; and NCDBBR, NCD supplemented with berberine. Different letters on the right side of the bars in **(B)** indicate significant differences. *p < 0.05; ***p < 0.001.

High-fat diet significantly alters the host gut microbiota structure and metabolism (Leone et al., 2015; Lin et al., 2019; Wang Y. et al., 2019). To clarify whether berberine alleviates liver inflammation and fat accumulation in mice by relieving disruptions in the gut microbiota structure caused by HFD, a total of 5,235,550 high-quality 16S rRNA gene V4–V5 sequences were obtained from 30 mouse stool samples. To eliminate the influence of the sequencing read depth on subsequent interpretation of results, 84,044 sequences were randomly selected from each sample prior to analysis. A total of 11,300 OTUs with 97% similarity were obtained. Except for a few OTUs that contained 0.013 ± 0.002% of all the sequences used for analysis, the majority of OTUs were divided into 25 phyla, among which Bacteroidetes, Deferribacteres, Firmicutes, Proteobacteria, and Verrucomicrobia were the dominant phyla (Figure 7A). Treatment with compound antibiotics (HFDABT and HFDABTBBR groups) significantly increased the relative abundance of Proteobacteria in the fecal microbiota of mice (Kruskal–Wallis *H*-test, *p* < 0.05; Figure 7B), indicating that the compound antibiotics used in this study had the poorest bacterial inhibitory effect against Proteobacteria compared to that against other bacteria. HFD significantly increased the relative abundance of Firmicutes and Deferribacteres in the fecal microbiota of mice, but significantly decreased that of Bacteroidetes (Kruskal–Wallis *H*-test, *p* < 0.05; Figure 7B). Compared with that of the HFD group, the relative abundance of Firmicutes in the fecal microbiota of the HFDBBR group was significantly reduced (Kruskal–Wallis *H*-test, *p* < 0.05), but was still higher than that of the NCD group. By comparison, the relative abundance of Bacteroidetes was significantly increased, although it was still lower than that in the NCD group (Kruskal–Wallis *H*-test, *p* < 0.05; Figure 7B). In addition, the relative abundance of Proteobacteria and Verrucomicrobia was significantly increased in the fecal microbiota of the HFDBBR group compared with that in the NCDBBR group (Kruskal–Wallis *H*-test, *p* < 0.05; Figure 7B and Supplementary Figure 2).

Principal co-ordinates analysis based on the composition of fecal microbiota OTUs showed that antibiotic treatment (HFDABT and HFDABTBBR groups) and HFD significantly altered the composition of mice fecal microbiota (PERMANOVA, *p* < 0.05; Figure 7C). Antibiotic treatment (HFDABT and HFDABTBBR groups) significantly reduced the number of OTUs, Shannon index, and Simpson index observed in mice fecal microbiota (Kruskal–Wallis *H*-test, *p* < 0.05; Supplementary Figure 3). In contrast, HFD significantly reduced the number of OTUs observed in the fecal microbiota of mice, but significantly increased the Shannon and Simpson indexes (Kruskal–Wallis *H*-test, *p* < 0.05; Supplementary Figure 3). The number of OTUs, Shannon index, and Simpson index observed in the fecal microbiota of mice fed on HFD were significantly reduced after berberine intervention (Kruskal–Wallis *H*-test, *p* < 0.05). Berberine treatment did not significantly reduce the number of OTUs observed in the fecal microbiota of mice fed on a normal diet (Kruskal–Wallis *H*-test, *p* > 0.05). However, berberine treatment significantly reduced the Shannon and Simpson indexes in these mice (Kruskal–Wallis *H*-test, *p* < 0.05; Supplementary Figure 3).

To further analyze the significant effects of HFD and berberine intervention on the composition of mice fecal microbiota, we conducted a statistical analysis of the dominant bacteria at the genus level. The results revealed significant differences among the 46 dominant bacterial genera detected (Kruskal–Wallis *H*-test, *p* < 0.05). Among them, the fecal microbiota of mice fed on an HFD was significantly enriched for *Mucispirillum*, *Odoribacter*, *Roseburia*, *Adlercreutzia*, *Oscillospira*, *Psychrobacter*, *Dehalobacterium*, *AF12*, *Coprococcus*, *Ruminococcus*, and some unidentified genera (Kruskal–Wallis *H*-test with Welch’s *post hoc* test, *p* < 0.05). These bacteria did not show signs of significant enrichment in the mice fecal microbiota of the HFDBBR group. In contrast, *Parabacteroides*, *Trabulsiella*, *Sutterella*, *Butyricimonas*, *Eubacterium*, *Coprobacillus*, *Robinsoniella*, *Bacteroides*, *Clostridium*, *Akkermansia*, and *Proteus* were significantly enriched in the fecal microbiota of HFDBBR mice (Kruskal–Wallis *H*-test with Welch’s *post hoc* test, *p* < 0.05; Figure 7D).

**FIGURE 8:**
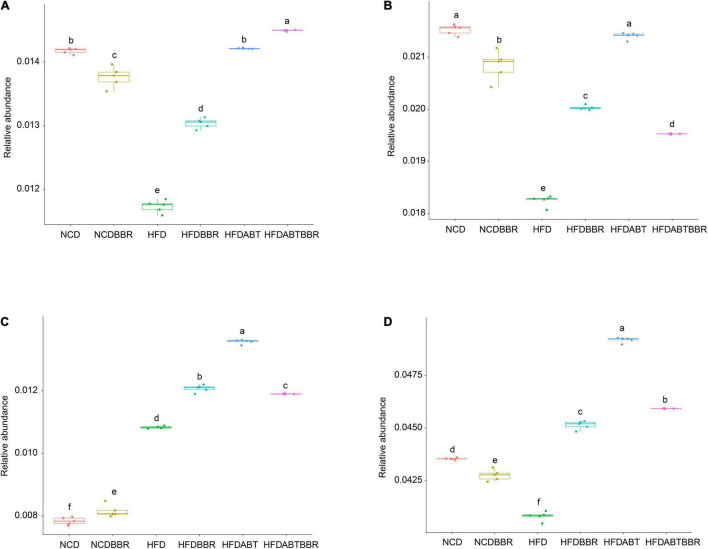
Relative abundances of genes in the butanoate **(A)**, propanoate **(B)**, and pyruvate **(C)** metabolisms, and all of the genes in the three metabolism pathways **(D)**. The functional profiles of gut microbiota were predicted using the phylogenetic investigation of communities by reconstruction of unobserved states. The different letters above the box indicate that there were significant differences between the data (*p* < 0.05).

Moreover, functional profiles of gut microbiota were predicted using the PICRUSt to analyze the relative abundance changes of metabolic genes producing short-chain fatty acids (SCFAs) among different groups. The relative abundances of genes in the butanoate, propanoate, and pyruvate metabolisms showed that the relative abundances of genes in the butanoate, and propanoate metabolisms were significantly reduced in the HFD group, and BBR treatment could alleviate the reduction, but it could not completely return to the control level (Figures 8A,B). The relative abundance of genes in the pyruvate metabolism was significantly increased in the HFD group compared with control (Figure 8C). Totally, HFD significantly reduced the relative abundance of genes involved in SCFAs in gut microbiota, and BBR treatment could alleviate the reduction (Figure 8D).

## Discussion

The inflammatory signaling pathway, wherein the transcription factor NF-κB participates, plays a central role in inflammation. As a cell signaling factor of the NF-κB signal transduction pathway, nitric oxide (NO) can participate in multiple physiological processes such as cellular communication, cell apoptosis, and the autoimmune response (Moncada and Palmer, 2001; Tripathi et al., 2007). When NO is expressed at high levels in cells, it can induce the production of inflammatory factors such as TNF-α, IL-1β, and IL-6. In turn, these pro-inflammatory factors can enhance the activity of iNOS and promote further production of NO, resulting in a vicious circle that intensifies the inflammatory response (Aktan, 2004; Nagy et al., 2007). *Cox-2* is an inducible gene that is either not expressed or expressed in very low levels in most tissues or cells under normal conditions. However, extracellular stimuli, such as endotoxins and *TNF-α*, can induce the expression of *COX-2* to help it exert its pathophysiological effects, which, in turn, can mediate an inflammatory cascade (Lanes et al., 2000). Reg3γ is an important factor in innate immunity and plays an important role in maintaining the dynamic balance among mammalian gut microbes and protecting the body from pathogen infection. Consistent with previous research (Xin et al., 2014; Wang Y. et al., 2019), we found that HFD successfully induced mice obesity and related metabolic disorders, as indicated by increased fat deposition, insulin resistance, and hepatic steatosis. These signs of metabolic syndrome were effectively improved after berberine treatment. In this study, berberine treatment not only reduced obesity but also considerably decreased the concentration of TNF-α, iNOS, Cox-2, and Reg3γ. The two common characteristics of NAFLD are lipid accumulation in liver cells and elevated production of pro-inflammatory factors by resident or infiltrating macrophages (Stojsavljevic et al., 2014). Liver macrophages, among which Kupffer cells derived from embryos are the most common, are dynamic participants in maintaining liver homeostasis (Guilliams et al., 2016). During the occurrence of NAFLD, liver macrophages are polarized, recruited, and proliferated (Meli et al., 2014). Macrophages are polarized across a broad spectrum, from a pro-inflammatory M1-like state, to an anti-inflammatory and pro-fibrogenic M2-like state (Menon and Sudheer, 2007). During infection with intracellular pathogenic microorganisms, macrophages typically show M1 polarization and express the corresponding pro-inflammatory factors to eliminate pathogenic microorganisms. However, in the presence of extracellular pathogenic microorganisms or allergens, macrophages undergo substitution activation (M2) and express Arg1 and other anti-inflammatory factors to promote tissue repair. Therefore, the precise regulation of macrophage polarization is very important for the treatment of metabolic diseases (Lin et al., 2019). The chemokines CCL2 and TNF-α, which are secreted by inflammatory liver cells, can cause Kupffer cells to proliferate and polarize toward M1 and express IL-6, IL-1β, TNF-α, and inducible nitric oxide synthase 2 (INOS2). Macrophages and T cells are important in the study of phagocytosis, cellular immunity, and molecular immunology (Gordon, 2016; Peterson et al., 2018). Moreover, these immune cells are closely related to the occurrence of liver fat metabolism diseases (Hill et al., 2014; Gordon and Pluddemann, 2017). Here, we observed an increased percentage of macrophages and T cells in the HFD group, which was suppressed by berberine treatment. In agreement with these findings, we observed that the expression of *Arg1* in HFD mice increased significantly, compared to that in NCD mice, and was efficiently reduced by berberine administration. Studies have shown that excessive calorie intake, increased fat accumulation, and fat toxicity can activate the production of effector molecules (cytokines) and cells that are mainly involved in innate immunity (Cai et al., 2005; Cani et al., 2007). These products can enhance chronic low-grade inflammation and induce the recruitment and activation of various mature immune cells (including mast cells, macrophages, and dendritic cells) and fat cells in metabolic tissues, particularly in adipose tissue, thereby further enhancing the inflammatory response (Lumeng and Saltiel, 2011; Sell et al., 2012). In this study, we found that an HFD hindered fat metabolism in mice livers and increased the inflammatory response. After berberine intervention, these phenomena were significantly improved.

As a “second genome” for modulating the health phenotype of the superorganism host, gut microbiota are closely associated with host nutrition, metabolism, and immunity (Jia et al., 2008; Jin et al., 2020). It has been reported that an HFD disrupts gut microbiota in two ways: via diminishing the levels of gut barrier-protecting probiotics such as Bifidobacteria and promoting the growth of endotoxin producers. These changes can result in high levels of lipopolysaccharide (LPS) in the host blood, thereby causing inflammation, and consequently, obesity and insulin resistance (Cani et al., 2007). Studies have reported that the antibacterial activity of berberine in the intestine is related to its anti-obesity effects. Berberine can reduce the entry of intestinal endotoxins into the blood circulation, thereby reducing the level of low-grade inflammation and preventing the development of metabolic diseases (Zhang et al., 2012). Similarly, our results showed that the LPS producers *Mucispirillum* and *Psychrobacter* (Smith et al., 2017; Herp et al., 2019) were significantly enriched in the HFD group, but not in the HFDBBR group. This result suggested that berberine might play a role in suppressing the inflammatory response by reducing the content of LPS in the blood. It has been shown that *Adlercreutzia* is a marker of abnormal lipid metabolism (Wei et al., 2018). In addition, a multi-omics analysis of patients with liver steatosis revealed that steatosis is significantly negatively correlated with *Coprococcus* (Alferink et al., 2021). Moreover, it has been reported that *Ruminococcus gnavus* plays an important role in the upregulation of oxidative stress-related gene pathways in the gut microbiota of patients with inflammatory bowel disease (Hall et al., 2017). Our research showed that these bacterial groups were significantly enriched in the HFD group, but not in the HFDBBR group. These findings suggest that berberine can improve lipid metabolism and inflammation by regulating the gut microbiota. Studies have shown that *Parabacteroides* can significantly improve obesity, insulin resistance, lipid metabolism disorders, and NAFLD symptoms in HFD-induced obese mice and in an ob/ob obesity mouse model (Wang K. et al., 2019). Furthermore, it has been reported that *Sutterella* in the intestine of patients with constipation-autistic spectrum disorder is reduced significantly (Dan et al., 2020). Additionally, it has been demonstrated that *Bacteroides* regulates liver lipid metabolism and reduces NAFLD by activating the Bacteroides-folate-liver axis pathway (Qiao et al., 2020). *Akkermansia muciniphila* can ameliorate the negative effect of interferon-γ on glucose tolerance, which is negatively related to obesity, diabetes, cardiovascular diseases, and low-grade inflammation (Greer et al., 2016). We found that these bacterial species were significantly enriched in the HFDBBR group, which indicated that berberine could significantly improve obesity, insulin resistance, lipid metabolism disorders, and NAFLD symptoms.

Studies have shown that SCFA-producing bacteria are beneficial to the host, as they protect the mucosa from pathogen-induced damage, supply colonocyte nutrients, and mitigate inflammation (Maslowski et al., 2009; Filippo et al., 2010). Together with preventing HFD-induced insulin resistance, berberine also significantly increases the abundance of SCFA-producing flora and SCFA levels in the rat intestines (Zhang et al., 2012). Our results showed that the SCFA producers *Butyricimonas*, *Eubacterium*, and *Clostridium* (Sakamoto et al., 2014; Xie et al., 2017; Mukherjee et al., 2020) were significantly enriched in the HFDBBR group, suggesting that SCFA-producing bacteria likely play an important role in the efficacy of berberine. Thus, we infer that the selective modulations of specific gut microbial phylotypes, particularly the enrichment of SCFA-producing bacteria, may participate in berberine-mediated alleviation of metabolic diseases in the host. However, some SCFA-producing bacteria, such as Roseburia, Lachnospiraceae unidentified species, Coprococcus, and Clostridiales unidentified species, were also enriched in the group of HFD. Although the results of functional prediction showed that the relative abundance of SCFA-producing genes in gut microbiota were significantly reduced in the HFD group, and BBR treatment could alleviate the reduction, whether berberine can increase the production of SCFA by regulating gut microbiota needs to be determined by measuring the content of fecal SCFA in the future.

Notably, previous studies have shown that *Odoribacter splanchnicus* is negatively correlated with obesity (Etxeberria et al., 2017), *Roseburia* is negatively correlated with the risk score of healthy high-risk gene carriers (Imhann et al., 2018), and *Oscillospira* is negatively correlated with body mass index and inflammation (Konikoff and Gophna, 2016). In a study demonstrating that sucralose alters the gut microbiota of mice and promotes liver inflammation, the abundance of *Dehalobacterium* was significantly reduced (Bain et al., 2017) and *AF12* was related to the beneficial effects of fecal bacteria transplantation (Lai et al., 2018). The baseline abundance of *Coprobacillus* correlated with COVID-19 severity (Zuo et al., 2020). *Proteus* can induce inflammatory responses *in vivo* and *in vitro* and may play a key role in the pathogenesis of Crohn’s disease (Zhang et al., 2020). *Trabulsiella* and *Robinsoniella* are the most abundant species in infants delivered by cesarean section and cecal contents and mucosa, respectively (Linnenbrink et al., 2013; Kuang et al., 2016). However, we found that *Odoribacter, Roseburia, Oscillospira, Dehalobacterium*, and *AF12* were significantly enriched in the HFD group, whereas *Coprobacillus, Proteus, Trabulsiella*, and *Robinsoniella* were significantly enriched in the HFDBBR group. The reasons for this need to be further explored. At the phylum level, significant differences in the relative abundance of Firmicutes and Bacteroidetes in gut microbiota have been observed between host groups with obesity and lean phenotypes (Turnbaugh et al., 2006). In a rat study, significantly higher abundances of the Actinobacteria and Verrucomicrobia phyla were observed in the HFD group than in the NCD group, but this trend was completely reversed by berberine co-administration (Zhang et al., 2012). Similarly in this study, we found that HFD significantly increased the relative abundance of Firmicutes and Deferribacteres in the fecal flora of mice, but significantly reduced Bacteroidetes abundance. Furthermore, the relative abundance of Firmicutes in the fecal flora of mice in the HFD group was significantly reduced after berberine intervention, but was still higher than that in the normal control group. By comparison, the relative abundance of Bacteroidetes in the fecal flora was significantly increased, but was lower than that in the normal control group. A possible explanation for these observations may be that the Bacteroides phylum is positively correlated with LDL and HDL cholesterol levels, whereas the Firmicutes phylum is negatively correlated with total cholesterol and LDL and HDL cholesterol levels (Harata et al., 2017). This indicates that berberine intervention improves lipid metabolism. Previous studies on the Shannon–Wiener diversity index have revealed that berberine significantly decreases the bacterial diversity of the gut microbiota in both NCD- and HFD-fed rats (Zhang et al., 2012, 2015). However, our results indicated that HFD significantly increased the Shannon and Simpson indexes of the fecal flora of mice, but significantly reduced the number of OTU. These findings demonstrated that the composition of the fecal microbiota of mice treated with berberine and HFD was closer to that of mice fed on a normal diet. Furthermore, our study indicated that berberine can improve the gut microbiota disorder caused by HFD to some extent.

Although high-throughput sequencing of 16S rRNA gene has been widely used to analyze microbiota composition in various environments (Wu et al., 2017; Gong et al., 2021), it is difficult to distinguish microorganisms into species or strain level, which hinders a more detailed analysis of the function of gut microbes at species or strain level. Additionally, it is important to further exploit the gut microbiota-derived metabolites of berberine through metabonomics analysis. However, how to distinguish between gut microbiota-derived and host-derived metabolites is still challenging.

## Conclusion

Our findings showed that berberine could improve chronic inflammatory metabolic syndrome induced by HFD to some extent and effectively improved the metabolism of high-fat food in mice, which correlated with the gut microbiota structure. This study may improve our understanding of host-microbe interactions during the treatment of metabolic diseases and provide useful insights into the action mechanism of berberine.

## Data Availability Statement

The datasets presented in this study can be found in online repositories. The names of the repository/repositories and accession number(s) can be found below: https://bigd.big.ac.cn/gsa/browse/CRA004451, CRA004451.

## Ethics Statement

The animal study was reviewed and approved by the Animal Ethics Committee of the Qilu Normal University.

## Author Contributions

JJL, JLL, and HS designed the experiments. JJL, JLL, and SW performed the animal experiments. JJL and JN conducted gut microbiota analysis. JJL, CZ, JJ, GW, and HS measured biochemical data and conducted immunohistochemical analysis. JJL and HS wrote the draft of the manuscript. All authors revised and approved the final version of the manuscript.

## Conflict of Interest

JN is an employee of Guangdong Meilikang Bio-Science Ltd., China. The remaining authors declare that the research was conducted in the absence of any commercial or financial relationships that could be construed as a potential conflict of interest.

## Publisher’s Note

All claims expressed in this article are solely those of the authors and do not necessarily represent those of their affiliated organizations, or those of the publisher, the editors and the reviewers. Any product that may be evaluated in this article, or claim that may be made by its manufacturer, is not guaranteed or endorsed by the publisher.

## Supplementary Material

Supplementary Figure 1 Changes in body weight during the experiment **(A)** and blood glucose levels of mice at the end of the experiment that detected using insulin tolerance test (ITT) **(B)** and oral glucose tolerance test (OGTT) **(C)**. Different letters above the boxplots indicate significant differences.

Supplementary Figure 2 Heatmap profile of the dominant phyla in the gut microbiota in mice. HFD, high fat diet group; HFDBBR, HFD supplemented with berberine; HFDABT, HFD supplemented with antibiotics (by drinking freely); HFDABTBBR, HFD supplemented with berberine and antibiotics; NCD, normal chow diet; and NCDBBR, NCD supplemented with berberine.

Supplementary Figure 3 α-diversity changes of gut microbiota in mice fed different diets. HFD, high fat diet group; HFDBBR, HFD supplemented with berberine; HFDABT, HFD supplemented with antibiotics (by drinking freely); HFDABTBBR, HFD supplemented with berberine and antibiotics; NCD, normal chow diet; and NCDBBR, NCD supplemented with berberine. **(A)** Observed OTUs; **(B)** Shannon index; and **(C)** Simpson index. Different letters above the boxplots indicate significant differences.
