# Supplementary figures and images for: Berberine Relieves Metabolic Syndrome in Mice by Inhibiting Liver Inflammation Caused by a High-Fat Diet and Potential Association With Gut Microbiota
Source: Front Microbiol. 2022 Jan 12;12:752512. doi: 10.3389/fmicb.2021.752512 (PMC8790126; doi:10.3389/fmicb.2021.752512)

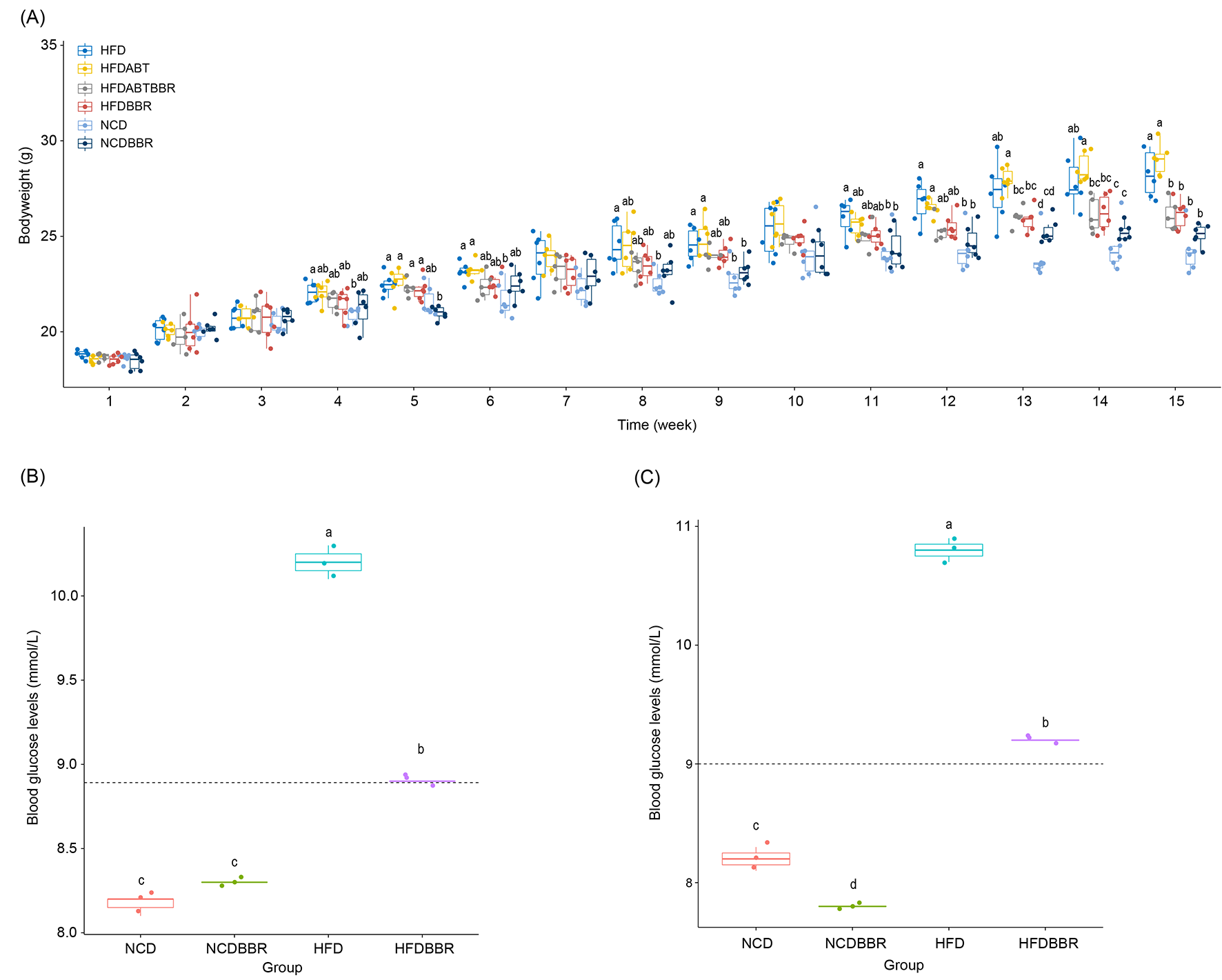

Supplement: Supplementary Figure 1 — Changes in body weight during the experiment (A) and blood glucose levels of mice at the end of the experiment that detected using insulin tolerance test (ITT) (B) and oral glucose tolerance test (OGTT) (C). Different letters above the boxplots indicate significant differences. [file Image_1.TIF]

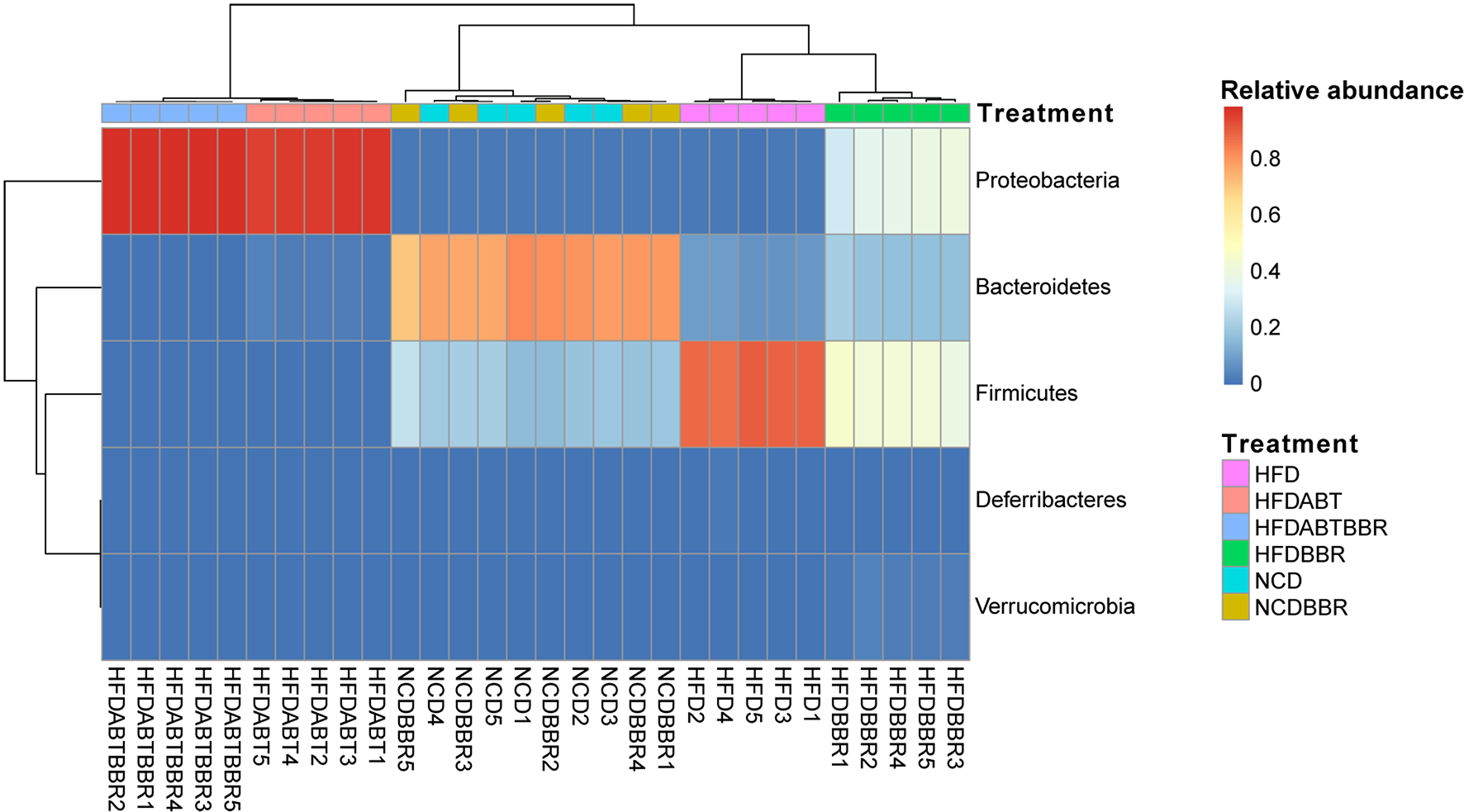

Supplement: Supplementary Figure 2 — Heatmap profile of the dominant phyla in the gut microbiota in mice. HFD, high fat diet group; HFDBBR, HFD supplemented with berberine; HFDABT, HFD supplemented with antibiotics (by drinking freely); HFDABTBBR, HFD supplemented with berberine and antibiotics; NCD, normal chow diet; and NCDBBR, NCD supplemented with berberine. [file Image_2.TIF]

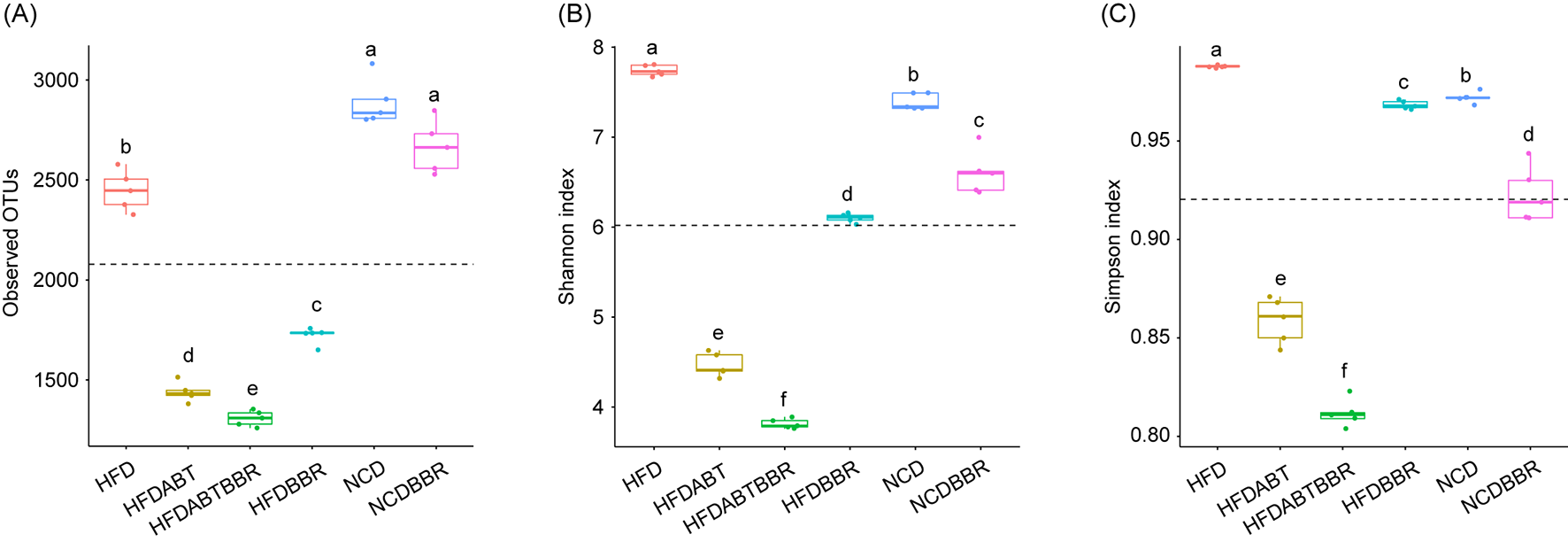

Supplement: Supplementary Figure 3 — α-diversity changes of gut microbiota in mice fed different diets. HFD, high fat diet group; HFDBBR, HFD supplemented with berberine; HFDABT, HFD supplemented with antibiotics (by drinking freely); HFDABTBBR, HFD supplemented with berberine and antibiotics; NCD, normal chow diet; and NCDBBR, NCD supplemented with berberine. (A) Observed OTUs; (B) Shannon index; and (C) Simpson index. Different letters above the boxplots indicate significant differences. [file Image_3.TIF]
