# Supplementary material for: Berberine Relieves Metabolic Syndrome in Mice by Inhibiting Liver Inflammation Caused by a High-Fat Diet and Potential Association With Gut Microbiota
Source: Front Microbiol. 2022 Jan 12;12:752512. doi: 10.3389/fmicb.2021.752512 (PMC8790126; doi:10.3389/fmicb.2021.752512)
Supplement: Supplementary file 5 [file Table_2.DOCX]

The merged DNA sequences were deposited in the Genome Sequence Archive with accession number CRA004451 (https://bigd.big.ac.cn/gsa/browse/CRA004451).

Download links of original microscopy images in the Figure 2, Figure 3, and Figure 4

**Figure 2**

HFD_5.0X

https://www.jianguoyun.com/p/Dct_F5sQ_bjdCRj9vIUE

HFD_10.0X

https://www.jianguoyun.com/p/DVK3lvwQ_bjdCRiGvYUE

HFD_20.0X

https://www.jianguoyun.com/p/DQExaiYQ_bjdCRiIvYUE

HFDABT_5.0X

https://www.jianguoyun.com/p/DSKev6kQ_bjdCRiLvYUE

HFDABT_10.0X

https://www.jianguoyun.com/p/DSVl9-MQ_bjdCRiPvYUE

HFDABT_20.0X

https://www.jianguoyun.com/p/DSM4cIgQ_bjdCRiRvYUE

HFDBBR_5.0X

https://www.jianguoyun.com/p/DavwSQMQ_bjdCRiTvYUE

HFDBBR_10.0X

https://www.jianguoyun.com/p/DbrFi_kQ_bjdCRiVvYUE

HFDBBR_20.0X

https://www.jianguoyun.com/p/DUkS8n8Q_bjdCRiavYUE

HFDABTBBR_5.0X

https://www.jianguoyun.com/p/DZO84yIQ_bjdCRievYUE

HFDABTBBR_10.0X

https://www.jianguoyun.com/p/DenT5p8Q_bjdCRijvYUE

HFDABTBBR_20.0X

https://www.jianguoyun.com/p/DcsLfdQQ_bjdCRixvYUE

NCD_5.0X

https://www.jianguoyun.com/p/DVc_WqsQ_bjdCRi7vYUE

NCD_10.0X

https://www.jianguoyun.com/p/DUSwN9wQ_bjdCRjCvYUE

NCD_20.0X

https://www.jianguoyun.com/p/DRl4vXMQ_bjdCRjFvYUE

NCDBBR_5.0X

https://www.jianguoyun.com/p/DQa0jS8Q_bjdCRjKvYUE

NCDBBR_10.0X

https://www.jianguoyun.com/p/DZDLh1EQ_bjdCRjOvYUE

NCDBBR_20.0X

https://www.jianguoyun.com/p/DWgf6rEQ_bjdCRjQvYUE

**Figure 3**

HFD+5.0X

https://www.jianguoyun.com/p/DYUzUckQqLndCRjTvYUE

HFD_10.0X

https://www.jianguoyun.com/p/DRMaOj4QqLndCRjtvYUE

HFD_20.0X

https://www.jianguoyun.com/p/DQD6XsQQqLndCRjzvYUE

HFDABT_5.0X

https://www.jianguoyun.com/p/Dco1AbQQqLndCRj1vYUE

HFDABT_10.0X

https://www.jianguoyun.com/p/DfEbN1wQqLndCRj2vYUE

HFDBBR_20.0X

https://www.jianguoyun.com/p/DS6_Tm8QqLndCRj3vYUE

HFDBBR_5.0X

https://www.jianguoyun.com/p/DZf2jacQqLndCRj6vYUE

HFDBBR_10.0X

https://www.jianguoyun.com/p/DfyHNUsQqLndCRj8vYUE

HFDBBR_20.0X

https://www.jianguoyun.com/p/DcruatsQqLndCRiDvoUE

HFDABTBBR_5.0X

https://www.jianguoyun.com/p/DUB99_0QqLndCRiEvoUE

HFDABTBBR_10.0X

https://www.jianguoyun.com/p/DZvPn-sQqLndCRiFvoUE

HFDABTBBR_20.0X

https://www.jianguoyun.com/p/DYFJPpIQqLndCRiIvoUE

NCD_5.0X

https://www.jianguoyun.com/p/DY4P-LUQqLndCRiLvoUE

NCD_10.0X

https://www.jianguoyun.com/p/DX3NgI4QqLndCRiPvoUE

NCD_20.0X

https://www.jianguoyun.com/p/Dcj38AgQqLndCRiTvoUE

NCDBBR_5.0X

https://www.jianguoyun.com/p/DYQnJBQQqLndCRiWvoUE

NCDBBR_10.0X

https://www.jianguoyun.com/p/DaQwgHsQqLndCRijvoUE

NCDBBR_20.0X

https://www.jianguoyun.com/p/DQ_y410QqLndCRikvoUE

**Figure 4**

HFD+5.0X

https://www.jianguoyun.com/p/DYrdcMIQubndCRjnzIUE

HFD_10.0X

https://www.jianguoyun.com/p/DSs9LpMQubndCRjtzIUE

HFD_20.0X

https://www.jianguoyun.com/p/DbZKTFUQubndCRjwzIUE

HFDABT_5.0X

https://www.jianguoyun.com/p/DbdLRtMQubndCRjyzIUE

HFDABT_10.0X

https://www.jianguoyun.com/p/DSCMkS8QubndCRjzzIUE

HFDBBR_20.0X

https://www.jianguoyun.com/p/DYPcob4QubndCRj0zIUE

HFDBBR_5.0X

https://www.jianguoyun.com/p/DXQKlMMQubndCRj2zIUE

HFDBBR_10.0X

https://www.jianguoyun.com/p/Da_y3KoQubndCRj3zIUE

HFDBBR_20.0X

https://www.jianguoyun.com/p/DVX3n-gQubndCRj6zIUE

HFDABTBBR_5.0X

https://www.jianguoyun.com/p/DRZq2SkQubndCRj7zIUE

HFDABTBBR_10.0X

https://www.jianguoyun.com/p/DWn8hUMQubndCRj8zIUE

HFDABTBBR_20.0X

https://www.jianguoyun.com/p/Dfytz8oQubndCRj-zIUE

NCD_5.0X

https://www.jianguoyun.com/p/DZ13xw8QubndCRj_zIUE

NCD_10.0X

https://www.jianguoyun.com/p/DdJV0EMQubndCRiBzYUE

NCD_20.0X

https://www.jianguoyun.com/p/Ddp5Nn4QubndCRiCzYUE

NCDBBR_5.0X

https://www.jianguoyun.com/p/Dd_fr4AQubndCRiDzYUE

NCDBBR_10.0X

https://www.jianguoyun.com/p/DdcQW6MQubndCRiEzYUE

NCDBBR_20.0X

https://www.jianguoyun.com/p/DadEIloQubndCRiFzYUE
